# Comprehensive dosimetric planning comparison for early‐stage, non‐small cell lung cancer with SABR: fixed‐beam IMRT versus VMAT versus TomoTherapy

**DOI:** 10.1120/jacmp.v17i5.6291

**Published:** 2016-09-08

**Authors:** Ilma Xhaferllari, Omar El‐Sherif, Stewart Gaede

**Affiliations:** ^1^ Department of Medical Biophysics Western University London ON; ^2^ Department of Oncology Western University London ON; ^3^ Department of Physics and Engineering London Regional Cancer Program London ON Canada

**Keywords:** VMAT, SABR, IMRT, helical tomotherapy, Acuros XB

## Abstract

Volumetric‐modulated arc therapy (VMAT) is emerging as a leading technology in treating early‐stage, non‐small cell lung cancer (NSCLC) with stereotactic ablative radiotherapy (SABR). However, two other modalities capable of delivering intensity‐modulated radiation therapy (IMRT) include fixed‐beam and helical TomoTherapy (HT). This study aims to provide an extensive dosimetric comparison among these various IMRT techniques for treating early‐stage NSCLC with SABR. Ten early‐stage NSCLC patients were retrospectively optimized using three fixed‐beam techniques via nine to eleven beams (high and low modulation step‐and‐shoot (SS), and sliding window (SW)), two VMAT techniques via two partial arcs (SmartArc (SA) and RapidArc (RA)), and three HT techniques via three different fan beam widths (1 cm, 2.5 cm, and 5 cm) for 80 plans total. Fixed‐beam and VMAT plans were generated using flattening filter‐free beams. SS and SA, HT treatment plans, and SW and RA were optimized using Pinnacle v9.1, Tomoplan v.3.1.1, and Eclipse (Acuros XB v11.3 algorithm), respectively. Dose‐volume histogram statistics, dose conformality, and treatment delivery efficiency were analyzed. VMAT treatment plans achieved significantly lower values for contralateral lung $V_{5\text{Gy}}(p\leq 0.05)$ compared to the HT plans, and significantly lower mean lung dose (p<0.006) compared to HT 5 cm treatment plans. In the comparison between the VMAT techniques, a significant reduction in the total monitor units (p=0.05) was found in the SA plans, while a significant decrease was observed in the dose falloff parameter, $D_{2\text{cm}}$, (p=0.05), for the RA treatments. The maximum cord dose was significantly reduced (p=0.017) in grouped RA&SA plans compared to SS. Estimated treatment time was significantly higher for HT and fixed‐beam plans compared to RA&SA (p<0.001). Although, a significant difference was not observed in the RA vs. SA (p=0.393). RA&SA outperformed HT in all parameters measured. Despite an increase in dose to the heart and bronchus, this study demonstrates that VMAT is dosimetrically advantageous in treating early‐stage NSCLC with SABR compared to fixed‐beam, while providing significantly shorter treatment times.

PACS number(s): 87.55.D, 87.55.dk, 87.55.kd

## I. INTRODUCTION

Cancer is the leading cause of death worldwide, with non‐small cell lung cancer (NSCLC) being the most dominant.^(1)^ In the treatment of early‐stage NSCLC, surgical resection is considered the standard of care.^(2)^ However, some patients are deemed medically inoperable due to age, decreased pulmonary reserve, cardiac function, or significant comorbidities.^(3)^ Medically inoperable patients, as well as patients unwilling to undergo surgery, have the option to be treated using stereotactic ablative radiotherapy (SABR). SABR is a hypofractionated technique where a very high ablative dose per treatment is delivered in few fractions, normally 3 to 8. Therefore, tumor conformality and sparing of normal tissue is increasingly crucial with SABR in comparison to conventional fractionation. SABR treatments are computed using multiple beam angles to achieve sharp dose gradients needed to spare healthy tissue. Outcome studies have shown SABR has an overall survival of 41.2% compared to 66.1% for patients who undergo lobectomy at five years; meanwhile, local control at three years has improved with SABR, 87.8%, compared to lobectomy resection, 85%.^(2)^

Although non‐coplanar, three‐dimensional conformal therapy (3D CRT) remains a popular technique for delivering SABR, intensity‐modulated radiation therapy (IMRT) has become increasingly popular due to the ability to improve dose conformality and reduce toxicities to normal tissue.^(4)^ There are various techniques available to compute IMRT: fixed beam (FB),^4^, ^5^ volumetric‐modulated arc therapy (VMAT),^(6)^ and helical tomotherapy (HT).^(7)^ FB involves holding the gantry fixed in each beam direction as each segment of the beam, formed using a multileaf collimator (MLC), is delivered. FB can be accomplished by step‐and‐shoot delivery (SS) and sliding window (SW). VMAT techniques deliver radiation using gantry rotation up to 360° around the patient while simultaneously varying gantry speed, leaf motion, and dose rate.^(8)^ Both FB and VMAT can be optimized using direct machine parameter optimization (DMPO) capable with the Pinnacle^3^ treatment planning system (Philips Medical Systems, Cleveland, OH) and Acuros XB (AXB) v11.3 dose calculation algorithm capable with the Eclipse treatment planning system (Varian Medical Systems, Palo Alto, CA). HT delivery is accomplished by synchronizing couch motion through the bore to the gantry rotation; intensity modulation is attained by a thin fan beam of various sizes and binary MLC^(9)^ and, most recently, with dynamic jaws.^(10)^

Lung SABR treatment plans consist of small fields with substantial heterogeneity from the high density tumor and the low density lung. Dose calculation algorithms available in commercial products vary in accuracy of dose computation.^11^, ^12^ The dose calculation algorithm available with Pinnacle^3^ and TomoTherapy treatment planning systems is collapsed cone convolution,^(13)^ and in Eclipse treatment planning system, AXB is employed.^(14)^

The goal of this retrospective planning study was to provide an extensive comparison of the various FB, VMAT, and HT techniques for delivering IMRT‐based treatment for early‐stage NSCLC patients with SABR. This study will conclude which technique and vendor provides the highest dosimetric benefit by comparing indices for the region of interest and organs at risk.

## II. MATERIALS AND METHODS

### A. Patient selection and contouring

A total of 10 patients with medically inoperable early‐stage NSCLC were enrolled in this retrospective planning study. These patients were chosen based on criteria of motion greater than 0.5 cm, and internal target volume (ITV) in the range of $4.4-53.1\;{\text{cm}}^{3}$, as typically observed in NSCLC SABR treatment cases. Patient‐specific characteristics, including staging, lesion location, and target volumes, are shown in Table 1.

Four‐dimensional computed tomography (4D CT) scans, reconstructed into 10 phases, were acquired for each patient using Varian's Real‐time Position Management (RPM) system (Varian

**Table 1:** General patient demographics.

*Patient*	*Stage*	*Tumor location*		*ITV size (cm^3^)*	*PTV size (cm^3^)*
1	T1bN0M0	RLL	Central	17.2	47.3
2	T1aN0M0	RLL	Peripheral	4.8	22.5
3	T2aN0M0	RLL	Central	22.6	58.8
4	T2aN0M0	RLL	Peripheral	35.2	78.0
5	T2aN0M0	RLL	Central	27.7	69.5
6	T2aN0M0	RLL	Peripheral	53.1	106.0
7	T1aN0M0	RML	Peripheral	16.2	42.8
8	T2aN0M0	RML	Central	48.8	103.8
9	T2bN0M0	LLL	Central	40.9	92.2
10	T1aN0M0	LUL	Peripheral	4.4	17.9

RLL=right lower lobe; RML=right middle lobe; LLL=left lower lobe; LUL=left upper lobe.

Medical Systems) in the Philips Brilliance Big Bore CT scanner (Philips Medical Systems). The gross target volume, (GTV), was contoured on each of the 10 respiratory phases and motion encompassing internal target volume, (ITV), was created by summing the 10 individual GTVs. Consecutively, the planning target volume (PTV) was created by adding a 5 mm expansion to the ITV in the untagged average 4D CT. Target volumes and contours of the critical structures were imported onto the untagged average 4D CT and employed for treatment planning across different techniques.

### B. Treatment planning

For each patient, eight treatment plans were optimized: three FB, two VMAT, and three HT plans, for a total of 80 treatment plans. A dose of 54 Gy in 3 fractions was prescribed for each patient. To ensure target coverage and provide normalization, 95% of the PTV must be covered by the prescription isodose (RTOG 0618).^(15)^

FB plans were computed by separate board‐certified dosimetrists who specialize in Eclipse and Pinnacle treatment planning. Prior to FB planning, the dosimetrists were instructed to use nine to eleven coplanar beams in each of their plans to have the highest quality plan attainable. For each patient, two SS plans, with a maximum allowed segments of 33 and 100 to represent low modulation (SS‐LM) and high modulation (SS‐HM), respectively, were retrospectively planned in Pinnacle^3^ v9.1 treatment planning system, and the dose was calculated using collapsed cone convolution. The SW plans were generated by a different board‐certified dosimetrist than the SS plans, using Eclipse treatment planning system; therefore, SW was composed of nine to eleven coplanar beams and did not have the same beam arrangement as SS. All the FB plans were recomputed using 10X flattening filter‐free (FFF) beams to optimize the efficiency of expected treatment delivery, and SW was recomputed using AXB dose algorithm version 11.3. Once recomputed, each of the plans was validated to ensure they are clinically acceptable, and if needed, the plans were reoptimized.

SA treatment plans were generated by employing clinically used Pinnacle host script via Pinnacle^3^ planning system with collapsed cone convolution, and RA treatment plans were computed with a clinically approved protocol in Eclipse planning system with AXB dose calculation algorithm. Two partial arcs were used depending on the location of the lesion in the lung to avoid overdosage to the contralateral lung. According to the clinical script, the SA plans consisted of two 225° beam arcs with the dimensions of $180.1^{\circ }-45^{\circ }$ clockwise and $45^{\circ }-180^{\circ }$ counterclockwise if the lesion was located in the right lung, or $315^{\circ }-179.9^{\circ }$ clockwise and $180^{\circ }-315^{\circ }$ counterclockwise if the lesion was located in the left lung. Whereas, the RA plans were computed using two 210° beam arcs with the dimensions of $180.1^{\circ }-30^{\circ }$ clockwise and $30^{\circ }-180^{\circ }$ counterclockwise if the lesion was in the right lung, and $330^{\circ }-179.9^{\circ }$ clockwise and $180^{\circ }-330^{\circ }$ counterclockwise if the lesion was situated in the left lung. Both partial arcs were generated using 10X FFF beam energy and a maximum 2400 MU/min dose rate.

All FB and VMAT techniques were prepared utilizing FFF beams to maximize efficiency in these hypofractionated deliveries. FFF beams allow for safe treatment delivery with dose rates up 2400 MU/min, significantly reducing treatment time.^16^, ^17^ Clinical assessment of utilizing FFF beams to treat early‐stage NSCLC patients with SABR have demonstrated early local control rates upwards of 89%.^(17)^

The three HT plans with varying beam fan width of 1 cm, 2.5 cm, and 5 cm (HT 1 cm, HT 2.5 cm, and HT 5 cm, respectively) were generated by a board certified dosimetrist using Hi·Art TomoPlan 3.1.1 (Accuracy Inc, Sunnyvale, CA). For the patients in this study, a 0.172 pitch and 1.3 modulation factor were used. All HT plans were designed using a 6X beam with 600 MU/min dose rate and optimized with inverse planning based on least squares optimization method. The dose was calculated by employing collapsed cone convolution algorithm.^9^, ^18^

Pinnacle, Eclipse, and HT treatment planning systems have different optimization methods, as well as varying cost functions. The planning constraints cannot be set the same between the different planning systems to achieve highest dose computation results within each treatment planning system. However, all plans computed in this study were clinically acceptable and satisfied SABR protocol.^19^, ^20^

### C. Plan comparison

The dose distribution from planning in all the different techniques and one set of contours were transferred to MiM v.5.6.5 (MiM Software Inc., Cleveland, OH) for analysis purposes. The independent software allows for consistent and unbiased plan evaluation based on dose‐volumetric histogram (DVH) parameters by using the same sampling algorithm. Parameters that characterize dose conformality, DVH statistics, and treatment delivery efficiency were obtained and compared. Further analysis to identify main difference amongst fixed beam, VMAT, and HT was performed by grouping the most clinically appropriate plans SS‐LM and SS‐HM plans, RA and SA VMAT plans, and HT 2.5 cm and HT 5 cm plans. SW and HT 1 cm were not included in the combined group analysis due to their inherit lack of efficiency.^9^, ^21^

#### C.1 Dose conformality

To evaluate dose falloff from the PTV to normal tissue, the maximum dose, at least 2 cm from the PTV, $D_{2\text{cm}}$, was calculated. For the PTV, the maximum and mean dose have been computed, and the conformality index was calculated for the 95% (${\text{CI}}_{95\%}$), 80% (${\text{CI}}_{80\%}$), and 50% (${\text{CI}}_{50\%}$) isodose levels according to the RTOG model defined by:

(1) $$CI_{RI}=\frac{V_{RI}}{TV}$$

where $V_{\text{RI}}$ represents the volume covered by the reference isodose and *TV* is the volume of the PTV.^(22)^

#### C.2 DVH statistics

The maximum point dose ($D_{\text{max}}$) to nearby critical organs at risk (OAR), such as the esophagus, spinal cord, heart, trachea, and proximal bronchus was compared amongst all patients. Lung toxicity parameters analyzed include the absolute volume of normal lung covered by 50% of the prescription or more ($V_{50\%}$), predictive of fibrosis,^(23)^ the mean dose to the normal lung (MLD), the normal lung receiving 5, 10, 20, 27 Gy or more ($V_{5\text{Gy}},V_{10\text{Gy}},V_{20\text{Gy}},V_{27\text{Gy}}$, respectively), and contralateral lung receiving at least 5 or 10 Gy ($V_{5\text{GyC}},V_{10\text{GyC}}$, respectively).

#### C.3 Treatment delivery efficiency

The intensity gradients in IMRT planning were acquired using multiple MLC‐based control points. Increased modulation induces increased MLC travel, potentially causing a devaluation of the MLC track, requiring more frequent replacement. The total MLC travel was compared between all fixed beam and VMAT plans. The total monitor units required for each treatment technique was analyzed to evaluate treatment efficiency. The treatment delivery time was simulated for each beam of the fixed beam and VMAT plans based on the dose rate for each segment. Plan automation, available with TrueBeam linear accelerators, was assumed. Meanwhile, the treatment delivery time in HT treatment plans was estimated based on the pitch and monitor units, available in the DICOM header of the radiation plan dose files.

### D. Statistical analysis

All dosimetric parameters compared in this study were summarized by their respective means and standard deviation (SD). Statistical analysis was performed in IBM SPSS v.20 (IBM SPSS Statistics for Windows, Armonk, NY) using Shapiro‐Wilk normality tests followed by one‐way analysis of variance. The data significantly deviates from a normal distribution if Shapiro‐Wilk test was less than 0.05, and the null hypothesis was rejected. If the distribution was considered not normal, a nonparametric test, Kruskal‐Wallis one‐way analysis of variance, was utilized to find significance, followed by a Wilcoxon‐Mann‐Whitney test to find between‐subject significance. Whereas, the data were considered normally distributed if the Shapiro‐Wilk test was greater than 0.05 and the null hypothesis was accepted. For normally distributed parameters, a one‐way analysis of variance (ANOVA) was computed to find significance, followed by a Tukey's post hoc test to check for between‐subjects significances.

## III. RESULTS

**Figure 1:**
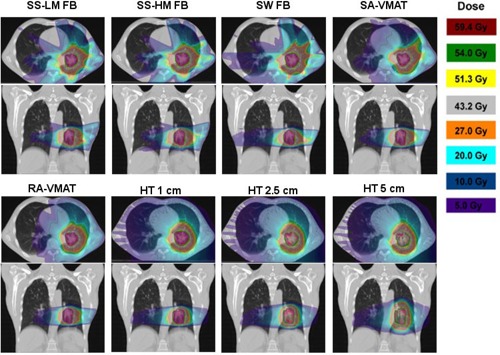
Dose distributions of the axial and coronal slice for each of the eight different planning techniques for Patient 4, from top left to bottom right: SS‐LM FB, SS‐HM FB, SW FB, SA, RA, HT 1 cm, HT 2.5 cm, and HT 5 cm.

**Figure 2:**
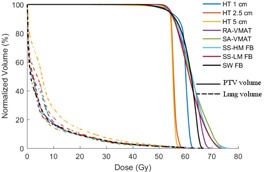
Cumulative DVH for Patient 4 for the PTV (solid lines) and the normal lung tissue (dashed lines) obtained from the eight techniques used. All plans are normalized such that 95% of the PTV receives 54 Gy or more.

Dose distribution for the eight various IMRT techniques compared in this study are displayed in Fig. 1 for one patient, and the corresponding DVH of the PTV and normal lung tissue, the lung tissue minus the ITV, are provided in Fig. 2. In the axial slice for all HT plans (Fig. 1), the contralateral lung is covered by the 5 Gy or higher isodose volume. As the width of the fan beam in HT increases to the 5 cm plan, an increase in the low dose spillage is noticed in the superior–inferior direction shown in the coronal slice. HT planning achieved dose homogeneity in the PTV surpassing other techniques, as can be found in the cumulative DVH (Fig. 2); however, for SABR treatment, dose uniformity and lack of hot spots within the target is not an essential priority for ablative radiotherapy. Although hypoxic regions are irradiated with all IMRT techniques, the increased heterogeneity within the PTV is regarded as clinically desirable, and provides the ability to deliver inherently higher doses to potential hypoxic regions.^24^, ^25^, ^26^

Table 2 summarizes the average and SD of the 10 patients for each of the parameters described in this study. Parameters for dose conformality, DVH related statistics, and treatment efficiency amongst different planning modalities are displayed, along with the between‐ and within‐subject significance. Although every plan met the SABR COMET criteria,^(19)^ all HT techniques showed a significance decrease in the PTV $D_{\text{max}}$ and mean dose compared to all other modalities in this study. This is further supported by the dose homogeneity seen in the PTV in Fig. 2. On the contrary, there was a significant increase in conformality index, ${\text{CI}}_{80\%},{\text{CI}}_{50\%}$, observed for the HT 5 cm plan compared to all other modalities, other than HT 2.5 cm for ${\text{CI}}_{50\%}$; RA&SA plans resulted in the most conformal to the PTV. A significant increase in contralateral $V_{5\text{Gy}}$ was observed for all HT plans (p=0.002) compared to SS and VMAT. A significant increase in mean lung dose was attained for the HT 5 cm plan (p=0.002). In both scenarios, RA&SA achieved the lowest values.

**Figure 3:**
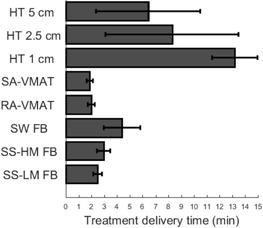
The mean estimated treatment delivery time for each treatment planning technique over all patients.

To further emphasize estimated treatment delivery time amongst all techniques, a significant increase was observed in all HT and SS plans compared to SA; however, a statistically significant difference is not found between RA&SA (p=0.393) (Fig. 3).

**Figure 4:**
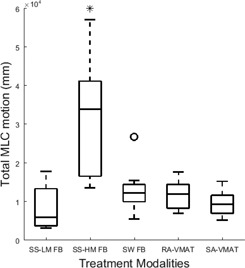
Box plot of the total MLC traveled in each plan in millimeters for all treatment modalities compared. For each plot, the median is displayed by the central line, the upper and lower border of the rectangle represent the 75th and 25th percentile or the interquartile range, and the whiskers represent the extreme data points not considered outliers. Outliers are illustrated by ‘o’ and significance is shown by ‘*.’

In the overall MLC travel comparison, SS‐HM required significantly more MLC motion than all other modalities compared, as shown in Fig. 4. SA resulted in the lowest MLC travel time and, therefore, least amount of potential degradation on the MLC track, compared to all other techniques, albeit, significance was not detected.

In the two VMAT techniques, RA displayed significantly superior $D_{2\text{cm}}$, dose falloff parameter, to SA (p=0.011), whereas total monitor units significantly increased (p=0.043). However, VMAT showed improved overall treatment quality and efficiency compared to all other modalities with SA achieving optimum efficiency.

Further analysis to identify main difference amongst fixed beam, VMAT, and HT was completed by grouping the SS‐LM and SS‐HM plans, RA and SA VMAT plans, and HT 2.5 cm and HT 5 cm plans (Table 3). SW and HM 1 cm treatment plans were not included in this analysis based on poor performance in efficiency parameters, while dose distribution was not improved, as observed in Table 2. A significant reduction in maximum dose to the spinal cord was observed in the VMAT plans (p=0.017) compared to the SS. Although not statistically significant, for the remainder of the parameters, a reduction was found between SS and VMAT (other than esophagus, bronchus, and heart). When comparing SS to HT, target conformality (${\text{CI}}_{95\%}$) significantly improved in the SS plans (p=0.015), at the cost of the target mean and maximum dose which was significantly reduced in the HT plans (p<0.001). Similarly, target mean and maximum dose (p<0.001) showed a significantly reduction in the HT plans compared to the VMAT plans, while all three target conformality parameters significantly improved with VMAT (p<0.05 for all). In the DVH parameters, maximum dose to the trachea and normal lung, $V_{10\text{Gy}}$ and $V_{5\text{Gy}}$, contralateral lung $V_{5\text{Gy}}$, and mean lung dose significantly reduced using both the SS and VMAT treatment planning compared to HT. These differences show that patients treated with SS or VMAT may be less susceptible to radiation‐induced lung toxicities to HT‐treated patients. Estimated treatment delivery time was significantly reduced with VMAT plans compared to all other techniques (p<0.001).

**Table 2:** Mean values + standard deviation of all parameters compared. A p‐value<0.05 determines significance.

		*SS‐LM*	*SS‐HM*	*SW*	*RA*	*SA*	*HT1 cm*	*HT 2.5 cm*	*HT 5 cm*	*p‐value*
PTV	$D_{\text{max}}(\text{Gy})$	73.8±4.1 ^f^, ^g^, ^h^	65.8±23.2 ^f^, ^g^, ^h^	71.7±3.3 ^f^, ^g^, ^h^	71.2±3.8 ^f^, ^g^, ^h^	74.3±2.9 ^f^, ^g^, ^h^	62.9±2.4 ^a^, ^b^, ^c^, ^d^	63±5.8 ^a^, ^b^, ^c^, ^d^, ^e^	64.1±7.4 ^a^, ^b^, ^c^, ^d^, ^e^	<0.001
	Mean dose (Gy)	62.6±19 ^f^, ^g^, ^h^	62.5±1.9 ^f^, ^g^, ^h^	62.5±1.4 ^f^, ^g^, ^h^	62±1.5 ^f^, ^g^, ^h^	61.4±1.2 ^f^, ^g^, ^h^	58.7±1.8 ^a^, ^b^, ^c^, ^d^, ^e^	58.6±3.4 ^a^, ^b^, ^c^, ^d^, ^e^	58.8±4.1 ^a^, ^b^, ^c^, ^d^, ^e^	<0.001
	$D_{2\text{cm}}$ (%)	58.6±8	58.4±6.8 ^c^	62.4±5.5 ^b^, ^d^	53.2±4.3 ^c^, ^e^, ^h^	60.5±6 ^d^	57.2±6	58.7±6.6	60.9±6.7 ^d^	<0.05
	${\text{CI}}_{95\%}$	1.28±0.08 ^h^	1.28±0.08 ^h^	1.3±0.09	1.23±0.09 ^f^, ^g^, ^h^	1.23±0.06 ^f^, ^g^, ^h^	1.37±0.13 ^d^, ^e^	1.35±0.14 ^d^, ^e^	1.43±0.19 ^a^, ^b^, ^d^, ^e^	<0.01
	${\text{CI}}_{80\%}$	1.96±0.23 ^h^	1.97±0.24 ^h^	1.88±0.18 ^h^	1.78±0.2 ^h^	1.89±0.19 ^h^	1.96±0.21 ^h^	2.07±0.31	2.35±0.47 ^a^, ^b^, ^c^, ^d^, ^e^, ^f^	<0.01
	${\text{CI}}_{50\%}$	4.75±0.77 ^h^	4.73±0.77 ^h^	4.75±0.72 ^h^	4.31±0.75 ^h^	4.56±0.84 ^h^	4.43±0.57 ^h^	4.92±0.83 ^h^	6.03±1.38 ^i^	<0.05
Cord	$D_{\text{max}}(\text{Gy})$	14.3±4	13.3±3.9	15.8±7.1	11.2±3.2	11.2±1.4	13.9±5.2	13.7±5.5	13.8±5.2	>0.05
Bronchus	$D_{\text{max}}(\text{Gy})$	13.2±9.3	13.4±8.7	15.5±10.8	16.2±9.2	17±11.1	17.2±11.9	18.7±13.9	19.6±14	>0.05
Esophagus	$D_{\text{max}}(\text{Gy})$	14.1±6.3	14.1±6.2	16.2±8.5	14.9±5.5	16.2±6.4	15.6±5.4	14.1±3.9	14.4±5.7	>0.05
Heart	$D_{\text{max}}(\text{Gy})$	14.5±6.4	15±6.7	17.9±7.6	17.1±7.2	18±6.4	19.2±10.9	18.4±11.3	17.6±10.2	>0.05
Trachea	$D_{\text{max}}(\text{Gy})$	2.4±5.2 ^h^	2±4.3 ^h^	1.5±2.7	1.1±2.1 ^h^	1.5±2.6	1.4±2.4	1.7±2.4	2.7±3 ^a^, ^b^, ^d^	>0.05
Total lung	MLD (Gy)	4.8±1.3 ^h^	4.8±1.3 ^h^	4.9±1.4 ^h^	4.7±1.2 ^h^	4.6±1 ^h^	5.1±1.3 ^h^	5.8±1.6	7.1±1.7 ^a^, ^b^, ^c^, ^d^, ^e^, ^f^	<0.01
	$V_{50\%}$ (cm^3^)	191±78	189±80	185±77	172±66	173±67	179±75	200±83	250±108	>0.05
	$V_{50\%}$ (%)	4.1±1.6	4±1.6	4±1.5	3.7±1.4	3.7±1.3	3.8±1.6	4.3±1.7	5.3±2	>0.05
	$V_{20\text{Gy}}$ (%)	6.3±2.3	6.2±2.2	6.2±2.4	5.9±2.2	6±2.1	5.9±2.3	6.7±2.6	8.3±2.9	>0.05
	$V_{10\text{Gy}}$ (%)	12.9±4.6 ^h^	12.8±4.4 ^h^	14.3±5	13.9±4.4	12.3±2.7 ^h^	14.1±4.8	16.7±6	20.7±7 ^a^, ^b^, ^e^	<0.01
	$V_{5\text{Gy}}$ (%)	23.5±6.9 ^h^	23.7±6.9 ^h^	24.7±7.7 ^h^	22.9±6.8 ^h^	22±6.2 ^g^, ^h^	29±8.2 ^h^	33.4±9.1 ^e^	40.8±11.8 ^a^, ^b^, ^c^, ^d^, ^e^, ^f^	<0.001
Cont. lung	$V_{10\text{Gy}}$ (%)	0.9±1.7	0.8±1.4	2.8±4.1	0.4±1	0.4±0.7	1.4±2	1.5±2.1	1.9±2.5	>0.05
	$V_{5\text{Gy}}$ (%)	7.9±6.6 ^f^, ^g^, ^h^	8.4±6.1 ^f^, ^g^, ^h^	10.8±8	6.6±7.6 ^f^, ^g^, ^h^	7.4±7 ^f^, ^g^, ^h^	18.9±10.9 ^a^, ^b^, ^d^, ^e^	19±11.2 ^a^, ^b^, ^d^, ^e^	21±15.2 ^a^, ^b^, ^d^, ^e^	<0.01
Efficiency	MLC Motion (cm)	837±509 ^b^	3170±1475 ^a^, ^c^, ^d^, ^e^	1279±563 ^b^	1184±341 ^b^	960±310 ^b^	N/A	N/A	N/A	<0.001
	Monitor Units	3823±792 ^b^, ^c^, ^d^, ^f^, ^g^	4946±1226 ^a^, ^c^, ^e^, ^f^	8288±3412 ^a^, ^b^, ^d^, ^e^, ^f^	4782±655 ^a^, ^c^, ^e^, ^f^	4023±678 ^b^, ^c^, ^d^, ^f^	11208±1510 ^a^, ^b^, ^c^, ^d^, ^e^, ^f^	7047±4407 ^a^	5457±3451 ^f^	<0.001
	Delivery time (min)	2.5±0.3 ^i^	3±0.5 ^i^	4.4±1.4 ^a^, ^b^, ^d^, ^e^, ^f^, ^g^	2±0.3 ^a^, ^b^, ^c^, ^d^, ^f^, ^g^, ^h^	1.9±0.2 ^a^, ^b^, ^c^, ^f^, ^g^, ^h^	13.2±1.8 ^a^, ^b^, ^c^, ^d^, ^e^, ^h^	8.3±5.2 ^a^, ^b^, ^c^, ^d^, ^e^	6.4±4.1 ^a^, ^b^, ^d^, ^e^, ^f^	<0.001

a Significance was found when variables are compared to

^a^ SS‐LM,

^b^ SS‐HM,

^c^ SW,

^d^ RA,

^e^ SA,

^f^ HT 1 cm,

^g^ HT 2.5 cm,

^h^ HT 5 cm;

^i^ Significance found in comparison to all techniques.

**Table 3:** Significance for each parameter studied between grouped SS, VMAT, and HT (significance identified when p<0.05).

		*p‐value*		
		*SS& VMAT*	*SS & HT*	*VMAT & HT*
PTV	$D_{\text{max}}(\text{Gy})$	0.957	<0.001	<0.001
	Mean dose (Gy)	0.213	<0.001	<0.001
	$D_{2\text{cm}}$ (%)	0.978	0.168	0.152
	${\text{CI}}_{95\%}$	0.058	0.015	<0.001
	${\text{CI}}_{80\%}$	0.117	0.055	<0.001
	${\text{CI}}_{50\%}$	0.176	0.083	0.003
Cord	$D_{\text{max}}(\text{Gy})$	0.017	0.507	0.323
Bronchus	$D_{\text{max}}(\text{Gy})$	0.611	0.218	0.742
Esophagus	$D_{\text{max}}(\text{Gy})$	0.686	0.993	0.755
Heart	$D_{\text{max}}(\text{Gy})$	0.152	0.552	0.552
Trachea	$D_{\text{max}}(\text{Gy})$	0.626	0.004	0.020
Total lung	MLD (Gy)	0.561	0.005	0.001
	$V_{50\%}$ (cm^3^)	0.779	0.369	0.113
	$V_{50\%}$ (%)	0.756	0.341	0.093
	$V_{20\%}$ (%)	0.918	0.247	0.119
	$V_{10\text{Gy}}$ (%)	0.990	0.002	0.002
	$V_{5\text{Gy}}$ (%)	0.898	<0.001	<0.001
Cont. lung	$V_{10\text{Gy}}$ (%)	0.778	0.091	0.044
	$V_{5\text{Gy}}$ (%)	0.204	<0.001	<0.001
Efficiency	MLC Motion	0.123	N/A	N/A
	Monitor Units	0.607	0.516	0.787
	Delivery time (min)	<0.001	<0.001	<0.001

## IV. DISCUSSION

In this study, all 80 IMRT plans generated conformal dose distributions and provided clinically acceptable plans according to the guidelines of RTOG 0618 and our in‐house protocol based on SABR‐COMET. Various IMRT planning techniques for treating SABR were compared to conclude which IMRT modality is propitious by proving the most favorable dose conformality, DVH parameters, and treatment efficiency. In this study, VMAT planning, RA and SA, provided for the optimal trade‐off in treatment efficiency and dose coverage.

Several other studies have investigated the role of different IMRT treatment techniques in the treatment of early stage NSCLC with SABR to reduce lung toxicities.^27^, ^28^ Kannarunimit et al.^(27)^ compared three SABR treatment techniques, robotic surgery, RA, and HT plan, for treatment of central lung with SABR. They concluded that HT and VMAT provided more efficient treatment delivery and higher target dose homogeneity while robotic surgery and VMAT provided a lower risk of radiation‐induced lung pneumonitis, with VMAT yielding the lowest risk in cases of large PTV coverage. During hypofractionated radiation treatment, sharp dose gradients outside the PTV are desirable; hot spots in the center of the PTV are invoked to aid a dose falloff outside the PTV,^24^, ^25^, ^26^ alluding to dose homogeneity parameters not being included in the study, where traditionally HT planning excels. The findings on the reduction of radiation‐induced pneumonitis during VMAT plans by Kannarunimit and colleagues were further supported by our study where RA and SA demonstrated the lowest risk of radiation pneumonitis by having the lowest MLD and $V_{20\text{Gy}}$ values. A significant reduction in MLD was found when grouping both VMAT techniques and comparing to HT (p<0.001); however, no significant difference was found between the two VMAT techniques.^(29)^ For further analysis of radiation‐induced lung toxicity, our study investigated the reduction in the risk of fibrosis amongst the different treatment planning techniques ($V_{50\%}$); VMAT achieved reduced $V_{50\%}$ values. However, the differences in $V_{50\%}$ were not found statistically significant.

Weyh et al.^(28)^ compared RA, HT, and fixed beam for SABR treatment to lesions in the peripheral lung to conclude RA and fixed beam plans were equivalent, but the reduction in treatment time with RA makes them more preferable. This study has supported their work and furthermore, our results demonstrate a decrease in all normal lung DVH parameters in RA and SA, albeit not significant. Weyh and colleagues executed their study in eight patients for a total of 24 treatment plans, whereas this study expands onto validating different treatment methods within FB, VMAT, and HT compromising of 80 treatment plans. Treatment plans in both the Weyh^(28)^ and Kannarunimit ^(27)^ studies were generated using traditional flattening filtered (FF) beams with analytical anisotropic algorithm (AAA) dose calculation algorithm. Meanwhile, FFF beams were utilized in all linear accelerator based plans. Studies have shown that FFF treatment planning provides equivalent dose distribution to FF beams while significantly reducing treatment delivery time and increasing dose distribution conformity.^(16)^ AXB dose calculation algorithm, computed in this study for Eclipse treatment planning in RA and SW plans, has been shown to generate treatment plans comparable to X‐ray voxel Monte Carlo (XVMC), developed by BrainLab (Brainlab AG, Feldkirchen, Germany).^(14)^ Moreover, AXB allows for faster computational time to XVMC, while maintaining higher accuracy when dealing with tissue heterogeneity in the lung compared to AAA.

An important parameter when considering IMRT treatment, degradation of the MLC carriage due to MLC motion required during treatment delivery. Even though a significance was only found when comparing each of the techniques to SS‐HM, within the VMAT techniques, there was a reduction in MLC motion for the SA in nine of ten patients compared to RA. SA‐based treatment planning could result in a longer lifespan of the MLC carriage.

The limited number of patients used in this study may have led to insufficient statistical power to show significance between some of the parameters analyzed. The statistical power achieved when measured using ANOVA repeated measures in G‐power v. 3.1.9.2 is 0.76.^(30)^

Although other various treatment modality comparison studies have been conducted for the treatment of NSCLC with SABR, to the best of our knowledge there are not any other studies comprehensively covering a wide range of different IMRT techniques from various commercial vendors. In this study, the most up‐to‐date treatment planning using FFF beams to reduce significantly treatment times was used for both fixed beam and VMAT planning.

## V. CONCLUSIONS

In the treatment of early‐stage NSCLC patients with SABR, this study has demonstrated VMAT treatment planning techniques to have the optimal trade‐off between dose conformality and sparing normal tissue, and treatment efficiency. Although all plans were clinically acceptable, VMAT outperformed HT in all parameters measured, and statistical superiority was observed in 11 parameters when comparing grouped VMAT and HT techniques. In the comparison between SS and VMAT techniques, an increase in dose to the heart, esophagus, and bronchus was observed, although insignificant. However, VMAT was dosimetrically advantageous in all other parameters, while providing significantly shorter treatment times than any other modality studied.

RA and SA VMAT techniques performed comparably; RA displayed significantly sharper dose falloff, while SA optimization was statistically more efficient.

## ACKNOWLEDGMENTS

Financial support from the Canadian Institute of Health Research (CIHR) Strategic Training Program in Cancer Research and Technology Transfer (CaRTT), the Ontario Institute for Cancer Research (OICR), and the Ontario Research Fund (ORF) is gratefully acknowledged.

## COPYRIGHT

This work is licensed under a Creative Commons Attribution 3.0 Unported License.

## Supporting information

Supplementary Material
