# Supplementary material for: Comprehensive dosimetric planning comparison for early‐stage, non‐small cell lung cancer with SABR: fixed‐beam IMRT versus VMAT versus TomoTherapy
Source: J Appl Clin Med Phys. 2016 Sep 8;17(5):329–40. doi: 10.1120/jacmp.v17i5.6291 (PMC5874107; doi:10.1120/jacmp.v17i5.6291)
Supplement: Supplementary file 1 — Supplementary Material [file ACM2-17-329-s001.docx]

Comprehensive dosimetric planning comparison for early stage non-small cell lung cancer with SABR: fixed-beam IMRT versus VMAT versus tomotherapy

**Ilma Xhaferllari^1, 2­^, B.Sc, Omar El-Sherif^1, 2^, M.Sc, Stewart Gaede^1, 2, 3^, Ph.D**

*^1^Department of Medical Biophysics, Western University, London, Ontario, Canada*

*^2^London Regional Cancer Program, London Health Sciences Centre, London, Ontario, Canada*

*^3^Department of Oncology, Western University and London Health Science Centre, London, Ontario, Canada*

*Ilma Xhaferllari*

*London Regional Cancer Program*

*A4-841 Department of Physics and Engineering*

*790 Commissioners Road East*

*P.O. Box 5165*

*London, Ontario N6A 4L6*

*E-mail:* [*ixhaferl@uwo.ca*](mailto:Ilma.Xhaferllari@lhsc.on.ca) *| Phone: 519-685-8600 ext. 54520*

Short Title: Dosimetric Planning Comparison for Early Stage NSCLC with SABR
